# A Modified Controlled Cortical Impact Technique to Model Mild Traumatic Brain Injury Mechanics in Mice

**DOI:** 10.3389/fneur.2014.00100

**Published:** 2014-06-18

**Authors:** YungChia Chen, Haojie Mao, King H. Yang, Ted Abel, David F. Meaney

**Affiliations:** ^1^Department of Bioengineering, University of Pennsylvania, Philadelphia, PA, USA; ^2^Bioengineering Center, Wayne State University, Detroit, MI, USA; ^3^Department of Biology, University of Pennsylvania, Philadelphia, PA, USA

**Keywords:** controlled cortical impact, mild traumatic brain injury, biomechanics, strain rate, glia reactivity

## Abstract

For the past 25 years, controlled cortical impact (CCI) has been a useful tool in traumatic brain injury (TBI) research, creating injury patterns that includes primary contusion, neuronal loss, and traumatic axonal damage. However, when CCI was first developed, very little was known on the underlying biomechanics of mild TBI. This paper uses information generated from recent computational models of mild TBI in humans to alter CCI and better reflect the biomechanical conditions of mild TBI. Using a finite element model of CCI in the mouse, we adjusted three primary features of CCI: the speed of the impact to achieve strain rates within the range associated with mild TBI, the shape, and material of the impounder to minimize strain concentrations in the brain, and the impact depth to control the peak deformation that occurred in the cortex and hippocampus. For these modified cortical impact conditions, we observed peak strains and strain rates throughout the brain were significantly reduced and consistent with estimated strains and strain rates observed in human mild TBI. We saw breakdown of the blood–brain barrier but no primary hemorrhage. Moreover, neuronal degeneration, axonal injury, and both astrocytic and microglia reactivity were observed up to 8 days after injury. Significant deficits in rotarod performance appeared early after injury, but we observed no impairment in spatial object recognition or contextual fear conditioning response 5 and 8 days after injury, respectively. Together, these data show that simulating the biomechanical conditions of mild TBI with a modified cortical impact technique produces regions of cellular reactivity and neuronal loss that coincide with only a transient behavioral impairment.

## Introduction

Traumatic brain injury (TBI) is a significant public health problem. Of the 1.7 million people diagnosed with TBI, about 75% of them are considered to have experienced mild traumatic brain injury (mTBI) (1). Although most mTBI patients do not have long term impairments (2) approximately 15% may experience symptoms for years, and these persisting deficits are a major contributor to the morbidity associated with the disease (3, 4). Current diagnosis for TBI relies on clinical assessment; (5–7) neuroimaging techniques such as computed axial tomography (CT), magnetic resonance imaging (MRI), and diffusion tensor imaging (DTI) are all used to better detect structural and functional changes in TBI patients (8–10). Diagnosis for mTBI is more difficult than either moderate or severe TBI, due in part to the rapid recovery of symptoms and the lack of a universal definition (11–14). However, once diagnosed, there are very few treatment options outside of rehabilitation; successful pre-clinical treatments have yet to translate to the clinical population (15).

**Figure 1:**
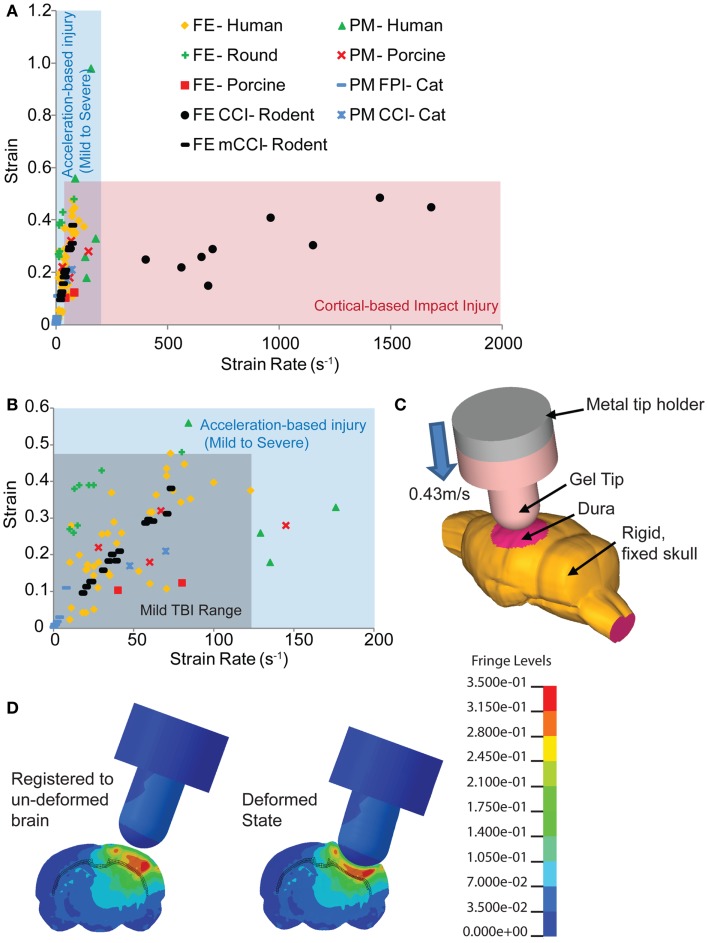
**Comparison of estimated strains and strain rates occurring in the brain from human-based and animal-based studies of brain biomechanics**. **(A)** The range of controlled cortical impact (CCI) brain injury extends over a much broader range of strain rates compared to those found in human and other animal TBI models. **(B)** A range of studies for the tissue loading conditions associated with mild TBI in humans provides a design corridor for modifying the cortical impact model to reproduce these mild TBI conditions in the mouse. **(C)** A finite element model of the mouse cortical impact model was used to study the tissue strain and strain rates that appear in the cortex and hippocampus when both impact depth and velocity are varied. The range of conditions for 0.43 m/s, 2 mm impact depth appears in **(B)** (black filled circles), while **(D)** shows the strain distribution of a mouse brain subject to these cortical impact conditions. References used: FE-Human (20, 39, 59–64), FE-Round (65), FE-Porcine (66); PM-Human (23), PM-Primate (24), PM-Porcine (43), FE CCI-Rodent (67, 68), PM CCI-Cat (69), PM FPI-Cat (45). FE, finite element; PM, physical model.

Creating an experimental model to reproduce faithfully human mTBI is challenging, yet can offer an important new tool to study mTBI in the laboratory. Re-creating the biomechanics of the tissue loading in the brain during injury is one key part of modeling mild TBI. In the past 10 years, biomechanics research successfully coupled finite element analyses (FEA) to existing TBI models and human accident reconstructions to produce a more detailed picture of the *in vivo* mechanical loading associated with concussions (16–22). These computational models analyzed the relationship between mechanical stress/strain and resulting structural/functional damage to tissue (23–36) to provide estimates on injury volume and mechanical injury thresholds for specific loading conditions (20, 37–42). In parallel, physical models of the brain also offered estimates of the tissue biomechanics associated with injury in the white matter (24, 25, 43). Among rodent mTBI models, strain rates of fluid percussion (FPI) and the dynamic cortical deformation injury models have similar ranges estimated in human mTBI (Figure 1) (37, 44, 45). Closed head injury rodent models may also yield strains and strain rates in the range of human TBI (46–55). Computational models of the brain mechanical response to either FPI and closed impact are difficult to develop, though, as some of the mechanical interactions in these models are difficult to quantify. Recently, a coronal rotational head injury in the rodent that uses accelerations scaled from human TBI was created and simulated in a two-dimensional finite element model (52, 56). However, this diffuse brain injury (DBI) model failed to show only axonal histopathological changes that are considered the hallmark for human mTBI; (57, 58) over 50% of the animals had hemorrhaging and lesions, pathologies that are not typical of mTBI.

With its precise impounder shape, impact depth, and controlled velocity, the controlled cortical impact (CCI) model is often the most direct model to study the biomechanics of TBI with a computationally based finite element model. The bulk of rodent TBI finite element models study the CCI technique (19, 67, 70, 71). However, even at slower speeds, CCI produces strain rates in the brain tissue of 400 s^−1^ and over (68) well over the higher strain rates of moderate to severe human TBI (Figure 1). At the more typical speeds, tissue strain rates range from 1150 to 1450 s^−1^ (67). Therefore, we revisited the biomechanics of traditional rodent CCI, altering it to produce injury conditions in the rodent that better approximate the brain tissue loading rates seen in human mTBI. To achieve this design goal, the impact velocity was reduced to achieve tissue strain rates more consistent with mild TBI, the cortical impact tip modified to distribute the strains throughout the cortex, and the impact depth adjusted accordingly. The combination of reducing impact velocity (strain rate) while maintaining impact depth (strain) created an injury pattern that included blood–brain barrier (BBB) opening, traumatic axonal injury, astrocyte reactivity, neuronal degeneration, and microglial activation without the significant hemorrhage and necrotic lesion often associated with cortical impact. Moreover, this modification achieves a slight, but temporary, behavioral deficit. The improved biomechanical fidelity of this model now provides a new tool to determine the tissue tolerance criteria associated with neuronal and glial changes in the brain after mTBI, as well as a scientific tool to compare the effects between a hemorrhagic and non-hemorrhagic injury.

## Materials and Methods

### Modified CCI device

The traditional controlled cortical impact (tCCI) for the mouse (72) produces a reproducible, graded injury that is influenced by the impounder shape, velocity, and depth of impact. The mild controlled cortical impact modified CCI (mCCI) model we designed retains many features of tCCI: an impounder strikes the exposed cortical surface, the impounder is returned to its original position, and the size of the craniotomy is within the range used in past cortical impact studies. Two main differences are the use of a rounded silicone tip (4 mm diameter; tip was made by injecting Sylgard 184 – mixed and vacuumed – into a 3-D printed mold) and adjusting the impact speed to reproduce the tissue loading biomechanics that occur in mild TBI. A rounded tip was chosen over the traditional, rounded cylindrical tip to minimize the hemorrhaging. A cylindrical shaped indentor has been shown to cause greater maximum principal strains and generate greater cortical hemorrhage than a rounded tip in traditional CCI (71, 73).

To develop a more precise estimate of impact velocity needed to achieve the tissue strain rates for mild TBI, we built a finite element model of the mouse brain and indentor used in the cortical impact technique. The finite element model (Figure 1C) incorporated a 4.5-mm craniotomy located on the left hemisphere, midway between bregma and lambda. The center of the impact was located at −2.5 mm bregma with a 20° angle of impact. An initial gap of 0.1 mm appeared between the gel tip and the exposed dura. To simulate injury, the rigid metal holder moved 2.1 mm downward into the model brain at 0.43 m/s. The finite element mouse brain model used was a previously validated model from Pleasant et al. (73). The silicone gel tip indentor was modeled using Ogden constitutive law available in LS-DYNA (LSTC, Livermore, CA, USA) with material properties from Lusardi et al. (74). For simplicity, only one combination of impact velocity (0.43 m/s) and impact depth (2 mm) was used. The range of resulting strain rates for elements within the hippocampus, cortex, and subcortical white matter of the ipsilateral hemisphere for a 2-mm, 0.43 m/s impact was 12–75 s^−1^ (black filled circles, Figure 1B). The larger impact diameter and increased impact depth creates a larger volume of tissue deformation (Figures 1C,D).

**Figure 2:**
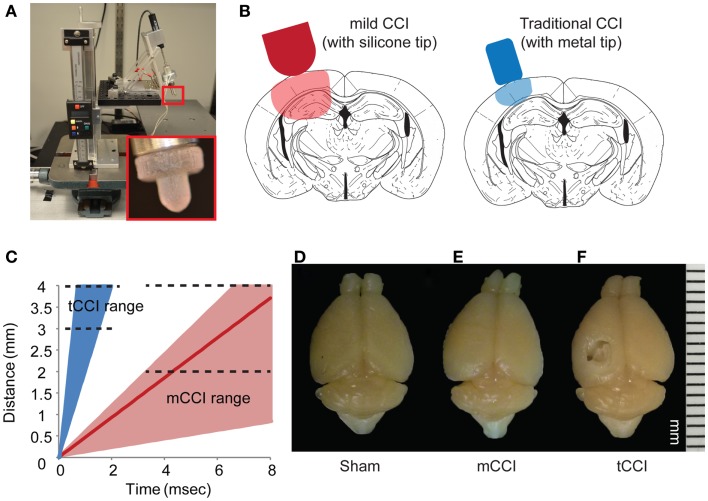
**Parameters of mild CCI**. The mild CCI (mCCI) uses a hemispherical silicone tip, actuated by a solenoid **(A)**. A potentiometer records the displacement of the impactor, while the angle and height of the impactor are fully adjustable. A comparison of displaced tissue volume between mild CCI (2 mm impact depth, 0.43 m/s impact velocity) and traditional CCI (1 mm impact depth, 4–6 m/s impact velocity) is shown in **(B)**. **(C)** Shows the range of impact speeds between tCCI (2.0–6.0 m/s) and the velocity range that would generate clinical TBI strain rates (17–104 s^−1^ for 0.1–0.6 m/s, respectively). The red line is the impactor speed used in this study (0.43 m/s for a strain rate of approximately 75 s^−1^). Brains perfused 8 days after a sham injury **(D)**, mCCI with 2 mm impact depth **(E)**, and tCCI with an impact depth and speed of 1 mm and 6.0 m/s, respectively **(F)**.

Using this as a guide, we constructed a modified cortical impact device on a mounting frame to minimize vibration during impact, and to align the indentor in the impact plane (Figure 2). A linear potentiometer (LP803-1, Omega, USA) measured the actuation of the solenoid and a custom Matlab (Mathworks, MA, USA) program controlled the solenoid and collected the potentiometer readings. The impactor was aligned 20° from vertical (measured with a digital angle meter), similar to other lateral CCI models. We also used a dwell time – defined as the duration over which the indentor is compressed into the brain – within the ranges (25–250 ms) for rodent CCI as cited in the literature (75). We could achieve higher impact speeds (4–6 m/s) if desired (Figure 2C), but chose to focus most of our efforts on the slower impact speed. At this lower impact speed, we saw no evidence of tissue necrosis 8 days after impact injury, unlike the extensive necrotic cavity that would appear after an impact using more commonly used impact speeds (4–6 m/s; Figure 2D).

### Cortical injury

All animal procedures were approved by University of Pennsylvania’s Institutional Animal Care and Use Committee (IACUC). Sixty 10–12-week-old male C57Bl6 mice (Jackson) were divided into sham and injured for three different timepoints. A 1-h timepoint to determine extravasation (sham = 3, injured *n* = 6); 24-h timepoint to determine early neurodegeneration (sham *n* = 5, injured *n* = 10 each); 8-day timepoint to determine longer term behavioral and histopathological changes (sham *n* = 17, injured *n* = 19).

On the day of injury, mice were anesthetized with isoflurane (5% induction, 2% maintenance in medical grade air) and placed in a stereotaxic frame on a heating pad to maintain body temperature. A 4-mm diameter craniotomy was produced on the left hemisphere, midway between bregma and lambda. CCI injuries were produced with a modified impactor at a speed of 0.43 m/s to a cortical impact depth of 2 mm (Figures 2B,C). The estimated center of the impact was −2.5 mm bregma. The solenoid has a full stroke length of 4 mm. The baseline point was set by lowering the impactor tip to the cortex with the solenoid in the fully actuated position and the linear stage position set to zero. Once baseline was set, the impactor was retracted and the impact depth was adjusted using 1.0 mm shims. We used a 2.0-mm impact depth for all studies presented in this paper. After injury, the cranial exposure was sutured close and the animals were placed in a warmed cage to recover until ambulatory. All animals survived the injury and surgery.

### Histology

Mice were anesthetized with an overdose of sodium pentobarbital. Animals were transcardially perfused with 30 mL of ice-cold phosphate-buffered saline (PBS) (pH 7.4) and then with 40 mL of ice-cold 4% paraformaldehyde. Brains were harvested, post-fixed overnight in 4% paraformaldehyde at 4°C, and then cryoprotected in 24% sucrose. Brains were mounted in Tissue-Tek^®^ and frozen in isopentane cooled with dry ice. Brains were cryosectioned at 20 μm. Sections were taken 500 μm apart starting from bregma −1.5 to −3.5 mm, therefore spanning the entire lesion area.

#### Blood–brain barrier compromise

For identification of extravasation, animals were injected with Evans blue (EB, 100μL of 4%) 15 min prior to the injury or sham surgery. One hour post injury, animals were perfused. Brains were removed, post-fixed, cyroprotected, and frozen. Equally spaced, serial tissue sections of the brain were mounted on slides and rinsed several times in PBS. The slides were coverslipped with ProLong Gold (Invitrogen, USA; #P36934). Sections were imaged with a Leica SP5 confocal microscope (Leica, Germany). The same laser settings were used with all sections.

#### Neuronal degeneration

Fluoro-Jade^®^ B (FJB) labeling detected degenerating neurons. Histological sections were rehydrated in graded ethanols (100, 95, 80, 70%; 2 min each) followed by distilled water. They were then incubated in a solution of 0.06% potassium permanganate on a rotating stage for 20 min. The sections were then rinsed in distilled water for 2 min and placed in fresh 0.0005% FJB solution made by adding 5 mL of a 0.01% stock solution of FJB to 95 mL of 0.1% acetic acid. After 30 min in the FJB staining solution, the sections were rinsed through three changes of distilled water for 1 min per change. Excess water was drained onto a paper towel, and the sections were then air dried in an oven at 37° for 30 min. The dry sections were cleared by immersion in xylene for at least 2 min before coverslipping with permount (Fisher Scientific, USA). The sections were viewed with a Leica SP5 confocal microscope (Leica, Germany). Positive FJB cell was determined with the same threshold across all images and the number of cells counted with ImageJ.

#### Astrocyte and microglial reactivity

Sections were rehydrated in 1 × PBS (3 × 5 min) and then incubated with 10% normal goat serum for 30 min with 0.1% triton-X. To measure astrocyte reactivity, sections were incubated overnight at room temperature in polyclonal anti-glial fibrillary acidic protein (GFAP) (Neuromics, MN, USA; #CH22102; 1:500). Images were taken with a Leica DFC340 FX (Leica, Germany) at three different locations for each brain region and the percent area above fluorescence threshold was averaged for each animal. The same settings were used for all sections. Microglial migration and reactivity as well as infiltrating macrophages were assessed using the polyclonal anti-Iba-1 (Wako, VA, USA; #019-19741; 1:1000). After rinsing with 1 × PBS, sections were then incubated with Alexa 546 conjugated secondary antibodies (Invitrogen, USA; #A-11035; 1:250) for 1 h at room temperature. Sections were mounted with ProLong Gold antifade (Invitrogen, USA; #P36934).

#### Axonal injury

Sections were dried and washed in 0.1 M TBS for 5 min. Sections were heated in 10 mM sodium citrate at 75°C for 30 min and allowed to cool to room temperature. Endogenous peroxidase activity was blocked with incubation in 3% hydrogen peroxide in methanol for 30 min with shaking. Sections were rinsed with running water for 10 min, after which they were placed in blocking buffer (0.1 M TBS and 2% FBS) for 5 min before blocking with 10% NGS for 30 min in 0.1 M TBS with 0.2% Triton-X. This was followed by overnight incubation at 4°C with anti-amyloid precursor protein (APP) (Invitrogen, USA; #36-3900; 1:1000). Sections were washed in 0.1 M TBS, placed in blocking buffer and then incubated in biotinylated goat anti rabbit secondary (Jackson Labs, #111-065-003; 1:250). Sections were washed and placed in blocking buffer again prior to incubation in Elite ABC (Vectastain, CA, USA; #PK-6100) for 30 min. Sections were washed and incubated in DAB (Vectastain, CA, USA; #SK-4100) for 5 min. Sections were washed in running water for 5 min and stained with Hematoxylin for 30 s. Sections were washed, dehydrated (alcohol 70, 80, 95% 2×, 100% 2×, xylene 2×), and coverslipped in Permount. Images were taken with a Leica DFC500 (Leica, Germany) and the number of varicosities were counted with ImageJ.

### Behavior tests

A series of behavior tests were conducted in the order that placed the test that required the least of amount of mouse handing first and the most stressful test last (Figure 8A). The rotarod training was conducted the day before the injury. On the day of injury, no behavioral test was given. On days 1–3 after injury, mice were tested on the rotarod; on days 4–5, mice were tested on the spatial object recognition (SOR); on days 7–8, mice were trained and tested in contextual fear conditioning (CFC).

#### Cortical motor function

The rotarod protocol was adapted from Oliveira et al. (76). In short, the rotarod apparatus (Med Associates Inc., VT, USA) has a 3.2-cm diameter rotating rod raised 16.5 cm above a platform and divided into five sections for testing multiple mice simultaneously. The rotarod gradually increased its rotation speed from 4 to 40 rpm over the course of 5 min. Latency to first fault (defined as the mouse riding with the platform in a single rotation) and fall time was recorded. Mice were trained on the rotarod at 4 rpm for 60 s 1 day before injury. Three trials a day were given during three consecutive days with an inter-trial interval of 1 h. Each trial started at the same time every day and ended when mice fell or when mice ran for 300 s.

#### Hippocampal function

The SOR protocol was adapted from in Oliveira et al. (77). In short, the experimental apparatus consisted of a gray rectangular open field (60 cm × 50 cm × 26 cm) with a visual cue placed on the arena wall. Prior to training, mice were handled for 1 min/day for 3 days. During the training day, mice received four 10 min training sessions. Between sessions, mice were placed back in their home cage for 3 min. During the first session, mice were habituated to the gray rectangular open field in the absence of objects, but with an internal cue on one of the four walls. For the next three sessions, mice were placed in the same box but now with two distinct objects. The objects consisted of a glass bottle (100 mL volume) and a metal tower (1.5625 in^2^). Mice were allowed to freely explore the environment and the objects for 10 min. After 24 h, mice were placed back in the rectangular environment for testing. The two objects were again present, but one of the two objects was now displaced to a novel spatial location. Mice were again allowed to freely explore the environment and the objects for 10 min. Both the object identity and spatial location was balanced between subjects. The response to changing the spatial location was assessed by comparing the mean time the mice spent exploring the objects (when mice were facing and sniffing the objects within very close proximity and/or touching them) belonging to each category (displaced and non-displaced) in the test session minus the mean time spent in contact with the same object category in the last training session. A positive value indicates recognition of the spatial change.

In the CFC test, mice were placed in a testing chamber (Coulbourn, PA, USA) on training day for 3 min. Two minutes and 28 s after the test started, mice were given a single 2 s, 1.5 mA shock. Twenty-four hours later, mice were placed in the same testing chamber and their percent freezing was calculated across the course of 5 min.

### Statistical analysis

All statistical tests were carried out using JMP10. The variance among groups was first tested for normality (Shapiro–Wilks test) and then equality (Bartlett test). When the variance among groups was not similar, the two-sample *t* test was used. Otherwise, a one way analysis of variance (ANOVA) was used. A linear regression model was used to look for significance between injured and uninjured groups. A repeated measures ANOVA was used in the rotarod behavior test.

## Results

### Mild controlled cortical impact induces blood–brain barrier extravasation

**Figure 3:**
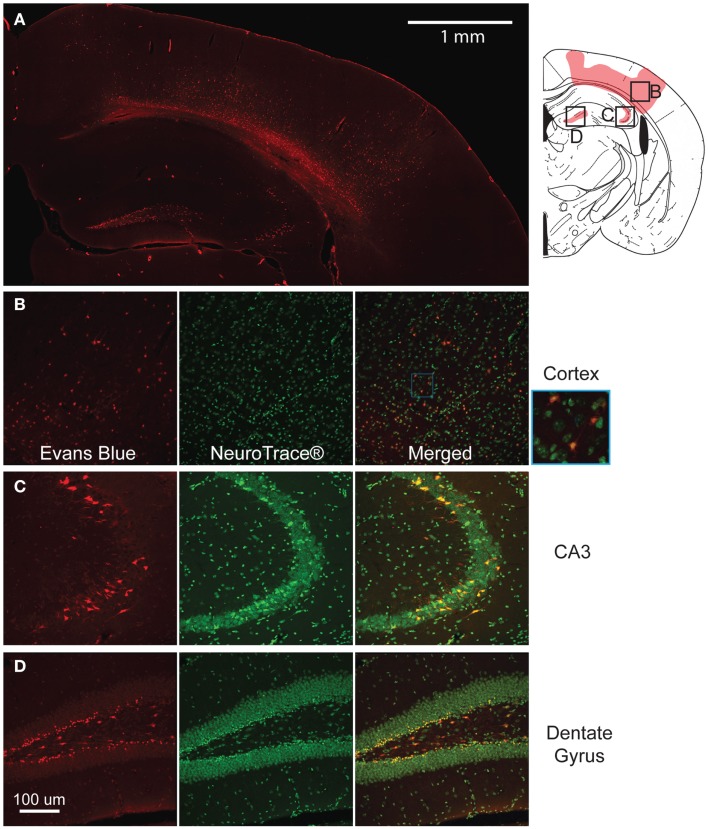
**Mild controlled cortical impact induces extravasation of the blood–brain barrier (BBB) at the injured region**. The pattern of extravasation is shown and pictorially depicted **(A)**. Co-labeling of EB and Neurotrace^®^ for the cortex **(B)**, CA3 **(C)**, and dentate gyrus **(D)** show that the EB positive cells are mostly neurons.

The modification of the CCI technique produced little to no visible lesion and no visible hemorrhaging immediately after impact, but EB staining appeared throughout the lesion site (Figure 3A). Although some variability in the EB staining intensity occurred across separate animals, there was no difference in the spatial distribution of EB staining. In the cortex, the staining formed an approximate hemispherical pattern. In comparison, a relatively higher concentration of EB staining appeared at the subcortical white matter directly below the impact site. In all animals tested, there was no staining in the cortical region directly contacting the impactor. In the hippocampus, the dorsal CA3 region (stratum pyramidale) and the dentate gyrus (some granular but mainly polymorphic layer) also showed staining. All injured animals had visible staining in the dentate across all five bregma sections analyzed. However, staining in the CA3 was only visible around bregma −1.5 and −2.0. No EB staining was seen in any of the sham animals (Supplemental Figure 1) or on the contralateral side.

At higher magnification, many of the cells in these regions showed positive staining and were closely associated with the vasculature. Triple labeling with GFAP, Neurotrace™, and EB showed that across all regions of interest (cortex, dentate gyrus, and CA3), a majority of EB stained cells were neurons (Figures 3B–D), indicating a preferential uptake of the dye by neurons.

### Mild controlled cortical impact induces neurodegeneration patterns that matches the EB patterns and persist for at least 8 days after injury

**Figure 4:**
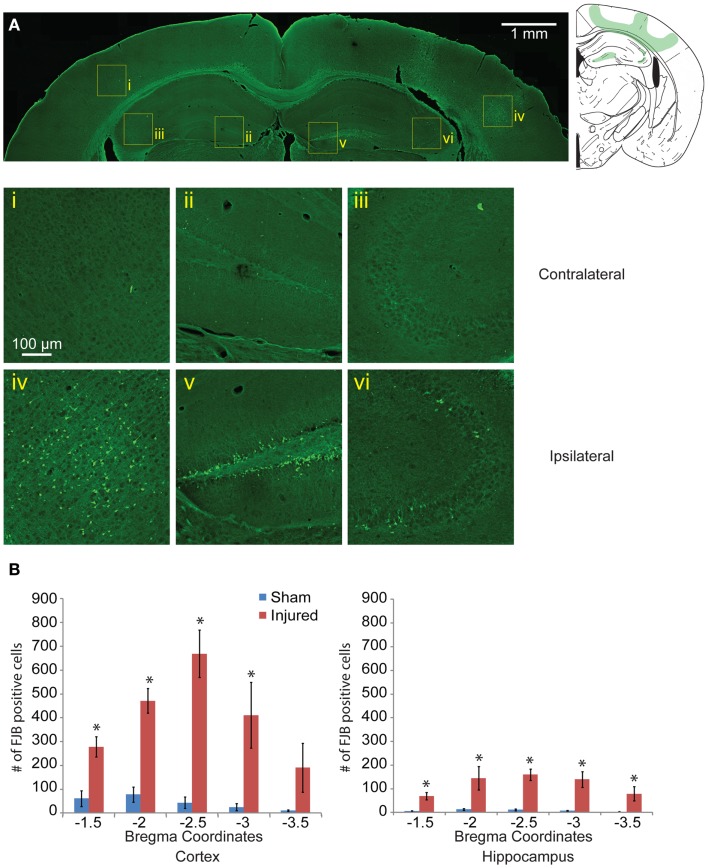
**Neuronal degeneration appears 24 h following mild controlled cortical impact**. Fluoro-Jade B (FJB) positive cells appeared in the cortex (iv), dentate gyrus (v), and CA3 (vi) on the ipsilateral side; no labeling was observed in the contralateral hemisphere [(i–iii), respectively] **(A)**. **(B)** The number of FJB positive cells in injured animals was significantly increased compared to sham in both the cortical and hippocampal regions for bregma sections −1.5 to −3.0 (cortex *p* < 0.001 for each section −1.5 to −3.0, for section −3.5 *p* = 0.0635, hippocampus *p* = 0.0113 for section −1.5, *p* < 0.001 for sections −2.0 to −3.5; *n* = 5 sham, *n* = 10 injured). Data are expressed as media ± SEM.

One day after injury, FJB staining showed a similar pattern to the EB staining (Figures 4Ai–vi), with a hemispherical degeneration pattern appearing immediately beneath the impact site. The cortical surface in direct contact with the indentor had no FJB staining. There was also elevated FJB staining in the subcortical white matter directly below the impact site. In the hippocampus, there were FJB positive cells in CA3 regions (stratum pyramidale) in bregma sections −1.5 and −2.0 but not in subsequent sections (Figure 4Avi). Staining in the dentate gyrus was apparent in all five bregma sections analyzed with the staining localized more to the polymorphic layer (Figure 4Av). Since FJB has been shown to co-label with astrocytes (78) sections were triple labeled with GFAP, FJB, and a fluorescent Nissl stain to determine which cell type(s) were FJB positive (data not shown). All FJB positive cells were positive for the Nissl stain and not GFAP. There was little to no FJB stained positive cells in the sham and on the contralateral side (Figures 4Ai–iii). In the sections imaged across the lesion, most showed significant levels of FJB positive cells between sham and injured animals (Figure 4B). Sections −1.5 to −3.0 showed significant FJB positive cells for both the cortex and hippocampus (cortex *p* < 0.001 for each section −1.5 to −3.0; hippocampus *p* = 0.0113 for section 1.5, *p* < 0.001 for sections −2.0 to −3.5). For section −3.5, the cortex trended toward significance at *p* = 0.0635.

**Figure 5:**
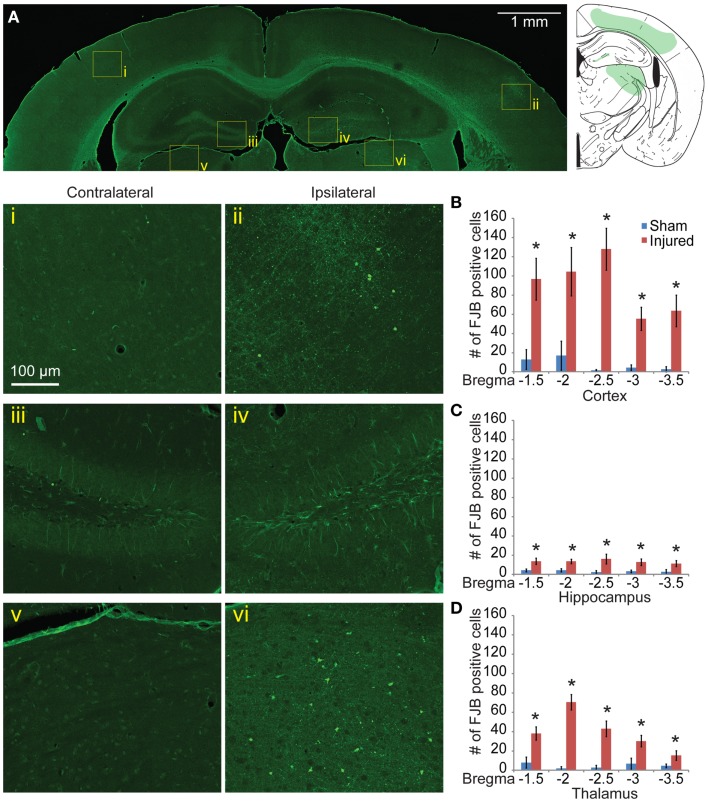
**Neuronal degeneration persists 8 days after mild controlled cortical impact**. Eight days after injury, Fluoro-Jade B staining was located in the cortex (ii), dentate gyrus (iv), and thalamus (vi) **(A)**. The contralateral regions did not show similar FJB staining (i, iii, v). **(B)** Degeneration in the cortex was still significantly elevated at 8 days compared to sham (*p* < 0.001 for all sections). **(C)** In the hippocampus, all sections show significant increase in FJB staining (bregma −1.5 *p* = 0.0105, −2.0 *p* = 0.0018, −2.5 *p* = 0.0102, −3.0 *p* = 0.0058, −3.5 *p* = 0.0338). **(D)** In the thalamus, all sections showed significant levels of FJB staining (−1.5 *p* = 0.0024, −2.0 and −2.5 *p* < 0.001, −3.0 *p* = 0.0081, −3.5 *p* = 0.0323). Samples sizes were *n* = 5 for sham, *n* = 11 for injured. Data are expressed as media ± SEM.

Neurodegeneration indicated with FJB staining was sustained 8 days post injury, although the staining was reduced relative to 24 h labeling. Although the level of positively stained cells in the cortex was reduced (Figure 5A), the pattern of FJB positive cells was still similar to that seen in at the 24-h time point. The number of FJB positive cells was still significant compared to sham controls (for cortical degeneration, Figures 5Ai,ii,B, *p* < 0.001 for all sections). Staining persisted in the CA3 and a significant retention of staining in the dentate gyrus appeared at this longer time point compared to sham (Figures 5Aiii,iv,C, bregma −1.5 = 0.0105, −2.0 *p* = 0.0018, −2.5 *p* = 0.0102, −3.0 *p* = 0.0058, −3.5 *p* = 0.0338). A new area of significant labeling appeared in the thalamus at this timepoint (Figures 5Av,vi,D, bregma −1.5 *p* = 0.0024, −2.0 and −2.5 *p* < 0.001, −3.0 *p* = 0.0081, −3.5 *p* = 0.0323). A Nissl stain revealed no obvious neuronal loss in these regions (data not shown).

### Mild controlled cortical impact induces axonal injury in the subcortical white matter

**Figure 6:**
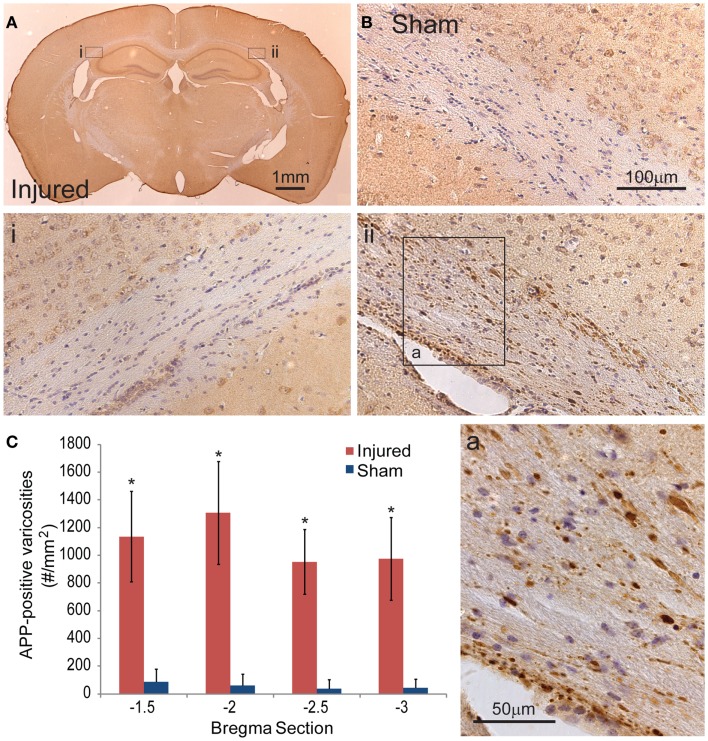
**Axonal injury appeared in the subcortical white matter after cortical impact injury**. **(A)** The amyloid precusor protein (APP) staining in the subcortical white matter on the ipsilateral hemisphere (ii) show a number of varicosities (insert a). Contralateral (i) regions between injured and sham brains **(B)** do not show similar axonal pathologies. **(C)** Quantification of the axonal varicosities shows significant number of APP varicosities near and at the epicenter of impact (*p* < 0.001 for all three bregma sections, *n* = 5 sham, *n* = 11 injured). Data are expressed as media ± SEM.

To document the level of axonal injury, tissue at the 24-h survival time were labeled for APP (Figures 6A,B). To quantify the level of axonal injury, we looked at the density of axonal varicosity in injured animals. A significant number of axonal varicosities were seen on the ipsilateral side in comparison to sham and the contralateral side (*p* < 0.001 for all sections analyzed) (Figures 6Ai,ii,B,C). The number of varicosities decreased with distance from the immediate impact site. Varicosities in areas other than the corpus callosum were not seen. The axonal varicosities present at 24 h were no longer apparent at 8 days (data not shown).

### Mild controlled cortical impact produces increase in gliosis that closely match EB and FJB patterns

**Figure 7:**
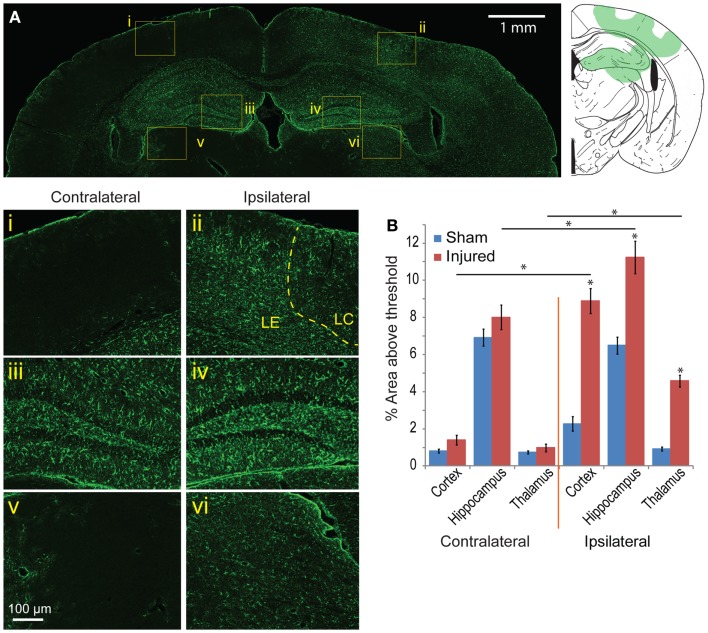
**Astrocyte reactivity is evident 8 days following mild cortical impact injury**. **(A)** In the ipsilateral cortex, there was no prominent glial scar (ii), although there were clear regions of astrocyte reactivity. The lesion center (LC) also exhibited less GFAP expression than the lesion edge (LE). (i) The contralateral cortex did not significant GFAP staining. In the hippocampus, there was slight increase of GFAP expression in the ipsilateral compared to contralateral hemisphere [(iv) versus (iii)], especially in the dentate gyrus. The ipsilateral thalamus also saw an increased GFAP expression [(vi) versus (v)]. **(B)** Comparing the contralateral and ipsilateral GFAP expression averaged showed that astrocyte reactivity was significantly elevated in the injured hemisphere across all five sections (*p* < 0.001 for each brain region); between sham and injured on the ipsilateral side, there was a significant increase for all three brain regions (*p* < 0.0001). Sample size *n* = 5 sham, *n* = 11 injured. Data are expressed as media ± SEM.

To determine the glia response to this injury model, astrocyte and microglia localization and expression were analyzed. On the cortex of the ipsilateral side, Iba-1 and GFAP staining patterns were similar to the EB and FJB staining patterns at 24 h and 8 day (Figures 7Ai–vi). GFAP expression immediately under the impact site was less than the region surrounding, a pattern similar to that seen in EB and FJB. Increased GFAP expression in the cortex 8 days after injury was more diffusely elevated, with a general increase in GFAP expression throughout the cortex, even at distal regions, compared to the contralateral side. GFAP expression in the hippocampus was also significantly elevated, especially within and surrounding the dentate gyrus (Figures 7Aiii,iv). There was also a significant increase in GFAP expression in the thalamus (Figures 7Av,vi). Across all five bregma sections imaged, the average GFAP expression was significantly elevated when compared with sham and the contralateral side (Figure 7B, *p* < 0.001 between cortex, hippocampus, and thalamus between sham and injured; *p* < 0.001 between contralateral and ipsilateral for cortex and thalamus). This suggested that regions with localized BBB extravasation regions produce degeneration, later eliciting an inflammation response.

**Figure 8:**
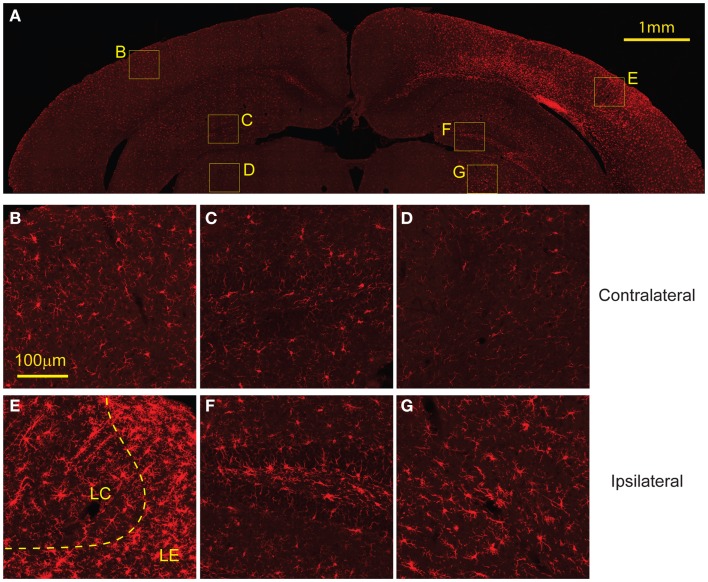
**Microglial activation occurs 8 days following injury**. **(A)** The ipsilateral cortex showed increased microglia migration, especially to regions that also showed FJB staining at 8 days. The ipsilateral cortex showed greater presence of activated microglia in the lesion edge (LE) compared to the lesion center (LC) **(E)**. **(B)** The contralateral cortex showed minimal microglia presence and no activated microglia. The ipsilateral hippocampus **(F)** and thalamus **(G)** show increased microglia migration compared to contralateral [hippocampus **(C)**, thalamus **(D)**] but both had less presence of activated microglia compared to the cortex. Data are expressed as media ± SEM.

Similarly, staining for microglia with Iba-1 showed increased presence of microglia in the same regions with elevated GFAP expression, FJB, and EB labeling (Figure 8A). In the cortex, the region directly subject to impact showed increased microglia migration and the presence of some activated microglia. However, regions surrounding the directly impact site showed a higher presence of activated microglia (Figure 8E). Increased microglia presence was also found in the ipsilateral hippocampus and thalamus compared to the contralateral side (Figures 8F,G). There was no evidence of activated microglia or increased microglia presence on the contralateral side (Figures 8B–D).

### Mild controlled cortical impact produces slight behavioral deficits in motor dependent tasks but not in hippocampal dependent tasks

**Figure 9:**
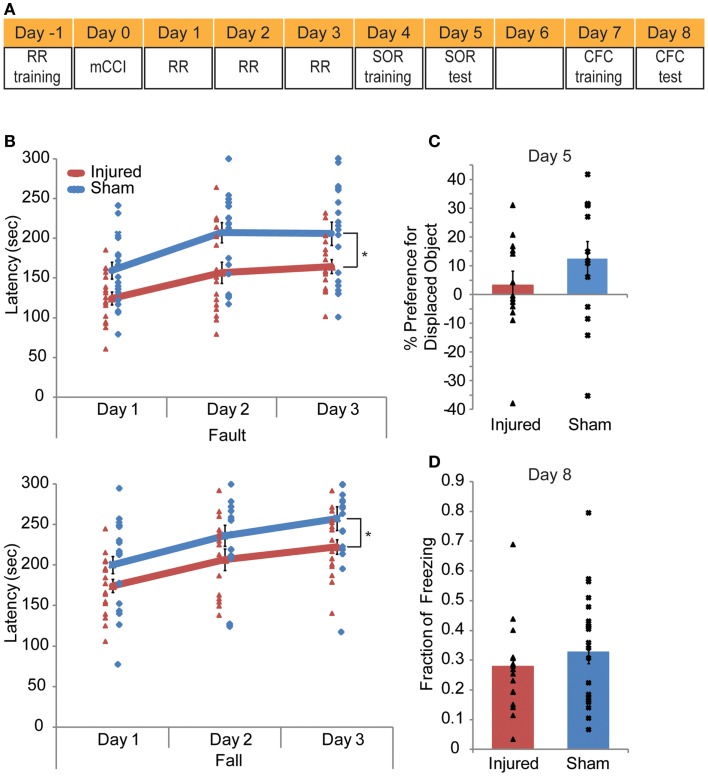
**Mild controlled cortical impact leads to a transient behavioral impairment**. A behavioral testing paradigm to examine both cortical and hippocampal function after impact is shown in **(A)**. Rotarod (RR) training occurred the day before injury; RR was given on days 1–3 post injury. Spatial object recognition (SOR) was implemented on days 4 (training) and 5 (test day). Contextual fear conditioning (CFC) was given on days 7 (training) and 8 (test). The animals were perfused on day 8 after CFC. The rotarod results **(B)** indicate injured animals faulted at lower speeds with significantly lower fault and fall latency times as determine by a repeated measures ANOVA (*p* = 0.0009 and 0.0017, respectively). In neither the SOR **(C)** nor the CFC **(D)** (*p* = 0.2345, *p* = 0.2182, respectively) did injured mice show significant level of altered behavior. Sample size *n* = 17 sham, *n* = 19 injured. Data are expressed as media ± SEM.

In all, mCCI produced very mild early behavioral deficits (Figure 9A). Injured mice showed vestibulomotor impairment after injury, as evident through the rotarod testing results. In repeated measures ANOVA showed significantly lower fault and fall times on the rotarod compared to sham mice (*p* = 0.0009, 0.0017, respectively, Figure 9B). The interaction between time and injury was not significant but was significant for time alone (*p* < 0.0001) showing that injured animals did not learn at a different rate than sham animals. At 4–5 days post injury, injured mice did not show a significant decreased preference for the displaced object than sham mice (*p* = 0.2345, Figure 9C) in the hippocampus dependent behavioral task, SOR. Additionally, the CFC task administered 7–8 days post injury also failed to elicit significant behavioral difference between injured and sham mice (*p* = 0.2182, Figure 9D). In all, mCCI produced early motor behavioral deficits.

## Discussion

The mild CCI model developed in this study produced early (24 h) and delayed (8 days) neurodegeneration, axonal injury in the subcortical white matter, and glial reactivity. These injuries appeared in conditions where the estimated tissue strain rates during impact were much less that the strain rates estimated for traditional cortical impact techniques (impact velocity: 4–6 m/s, strain rates: 400–1650 s^−1^). Early impairments in cortical motor function were the only behavioral deficits appearing in the mild CCI model. Later term measures of hippocampal function were not significantly different following injury, despite the brain showing broad areas of astrocyte and microglial reactivity. Together, these data show that re-creating the biomechanical conditions of mild TBI in the mouse brain led to an animal model of TBI that had very different histopathological and behavioral outcomes than the traditional cortical impact injury model.

Biomechanically, the mild controlled cortical impact studied herein shares some similarities to FPI injury. Both models have much slower loading rates than tCCI and produce injuries that are more in the range of human mTBI (Figure 1). While both models produce direct cortical deformation requiring craniotomies, loading on the brain between the two models is very different. FPI injury produces variable loading on the dura (79) whereas the indentor of a CCI provides a more consistent, repeatable deformation of the cortex. However, FPI injury offers a distinct advantage over cortical impact, as one can direct the injury to either a midline or lateral location (80). The midline FPI (MFPI) produces diffuse injuries, slight hemorrhages on the parieto-occipital cortex, corpus callosum, fimbria hippocampi, thalamus, and brainstem; high level injures produce similar but more extensive hemorrhaging patterns also encompassing the frontal, parietal, and occipital cortices (81, 82). Lateral FPI (LFPI) shares some features with the mild cortical impact technique developed in the study, since it produces focal injuries in addition to diffuse injuries. Moreover, mild LFPI produced BBB extravasation but the pattern was different than those of mCCI, perhaps due to the more widespread distribution of fluid across the cortical surface (78). While both mild FPI and mCCI produced axonal damage, increased gliosis, and neuronal cell loss, the largest difference between the two models is in the correlation of injury patterns between each of the histopathological stains. In mCCI, the regions of gliosis, degeneration, and BBB extravasations were very similar, something that has never been noted for mild FPI injuries.

The more diverse histopathology of mild controlled cortical impact, in the absence of contusion, offers a new opportunity to study the mechanisms of injury *in vivo* and determine how they correlate to the tissue biomechanics of mild TBI. For example, evidence for immediate compromise of the plasma membrane (i.e., mechanoporation) after injury can be explored with mCCI, significantly extending past studies of this primary mechanism of injury following either cortical impact or FPI injury (83). The model studied in this paper is not complicated by the presence of hemorrhage or tissue tears, which can also contribute directly to membrane compromise and neuronal degeneration. Owing to the viscoelastic properties of the plasma membrane, high strain rate loading may lead to a lower tolerance to mechanoporation than at lower strain rates, where the membrane may accommodate deformation more easily without failing. Similarly, the changes in glutamate receptor physiology – e.g., both the loss of the NMDA receptor magnesium block and the loss of AMPA receptor desensitization (62, 84–86) – could be examined in the new model where the biomechanical conditions of mild TBI are more controllable.

A second area of research enabled by this mCCI model is defining the mechanical tolerance of the brain to repetitive injury. Past work suggests that repeated insults of the same magnitude will lead to more prolonged neurological deficits and cellular changes (87) with these changes even more pronounced if the second injury is delivered within 1 week of the initial injury (50, 88–90). However, defining the mechanical tolerance to repetitive injury is not easily addressed using past models, primarily because it is difficult to precisely control each insult. Past repeated head injury models, which have typically been closed head impact or FPI cannot be easily analyzed computationally making it difficult to determine biomechanical thresholds to a subsequent injury. With the mechanical design of our technique, our cortical impact modifications appear ideally suited to test the effect of repeated, precise mechanical insults to the brain. We are especially encouraged that the transient behavioral deficits appear after even single mild, low velocity insult because this insult may allow one to explore if these deficits are either more severe or more prolonged after repeat insults, as suggested by past work (50, 88–90). In combination with finite element analysis of the tissue strains and strain rates that appear in areas of long term damage, we will likely start developing a more quantitative correlation among the biomechanics of injury, the tolerance of different brain regions to these injuries, and how these thresholds are modified with repetitive injury. In the long term, these correlations will be key in understanding protective strategies for the brain exposed to repeated insults, as well as providing more insights into the degenerating brain structure after repetitive insults of more complex origins.

## Conflict of Interest Statement

The authors declare that the research was conducted in the absence of any commercial or financial relationships that could be construed as a potential conflict of interest.

## Supplementary Material

The Supplementary Material for this article can be found online at http://www.frontiersin.org/Journal/10.3389/fneur.2014.00100/abstract
