# Supplementary figures and images for: A Modified Controlled Cortical Impact Technique to Model Mild Traumatic Brain Injury Mechanics in Mice
Source: Front Neurol. 2014 Jun 18;5:100. doi: 10.3389/fneur.2014.00100 (PMC4061598; doi:10.3389/fneur.2014.00100)

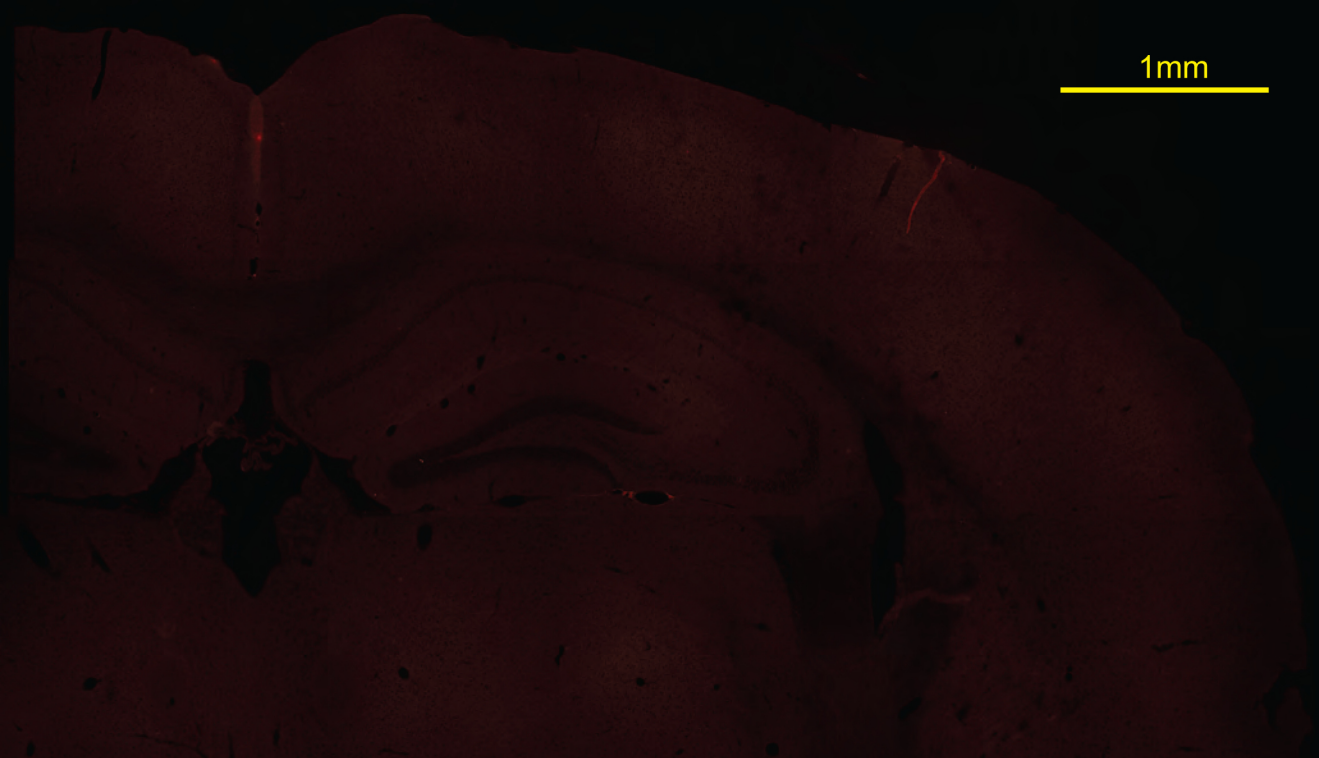

**Supplemental Figure 1. Sham animals show no EB staining on the ipsilateral side.**

Supplement: Supplementary file 1 [file DataSheet_1.PDF]
